# Perioperative corticosteroid administration: a systematic review and descriptive analysis

**DOI:** 10.1186/s13741-018-0092-9

**Published:** 2018-06-08

**Authors:** C. Groleau, S. N. Morin, L. Vautour, A. Amar-Zifkin, A. Bessissow

**Affiliations:** 1 Hematology Residency Program, McGill University, Montreal, Canada; 2 Department of Medicine, Division of General Internal Medicine, McGill University Health Centre, Montreal, Canada; 3 Department of Medicine, Division of Endocrinology, McGill University Health Centre, Montreal, Canada; 4 Medical library, McGill University Health Centre, Montreal, Canada

## Abstract

**Background:**

Perioperative administration of corticosteroid is common and variable. Guidelines for perioperative corticosteroid administration before non-cardiac non-transplant surgery in patients with current or previous corticosteroid use to reduce the risk of adrenal insufficiency are lacking. Perioperative use of corticosteroid may be associated with serious adverse events, namely hyperglycemia, infection, and poor wound healing.

**Objective:**

To determine whether perioperative administration of corticosteroids, compared to placebo or no intervention, reduces the incidence of adrenal insufficiency in adult patients undergoing non-cardiac surgery who were or are exposed to corticosteroids.

**Methods:**

We searched MEDLINE via Ovid and PubMed, EMBASE via Ovid, and the Cochrane Central Register of Controlled Trials, all from 1995 to January 2017.

**Selection criteria:**

We included randomized controlled trials (RCTs), cohort studies, case-studies, and systematic reviews involving adults undergoing non-cardiac non-transplant surgery and reporting the incidence of postoperative adrenal insufficiency.

**Data collection and analysis:**

Two authors independently assessed studies’ quality and extracted data. A descriptive and bias assessment analysis was performed.

**Results:**

Two RCTs (total of 37 patients), five cohort studies (total of 462 patients), and four systematic reviews were included. Neither RCT showed a significant difference in the outcome. This result was like that of the five cohort studies. The quality of the evidence was low.

**Conclusion:**

The current use of perioperative corticosteroid supplementation to prevent adrenal insufficiency is not supported by evidence. Given the significant studies’ limitations, it is not possible to conclude that perioperative administration of corticosteroids, compared to placebo, reduces the incidence of adrenal insufficiency.

**Electronic supplementary material:**

The online version of this article (10.1186/s13741-018-0092-9) contains supplementary material, which is available to authorized users.

## Background

It is increasingly common to encounter patients undergoing surgery who are on chronic corticosteroids or who have received large amounts of corticosteroids (Ergina et al. 1993). When evaluating the risk of adrenal insufficiency, it becomes a challenge for physicians to decide whether additional corticosteroid administration is required perioperatively.

Administration of stress-dose corticosteroids perioperatively for patients on long-term corticosteroid therapy began following the publication of two case reports in the 1950s. This happened despite the uncertainty regarding whether the patients in the case reports actually died of Addisonian crisis (Fraser et al. 1952; Lewis et al. 1953). Four decades later, a literature review demonstrated only three cases out of 57 in which death or hypotension could clearly be attributed to perioperative adrenal crisis in patients on long-term corticosteroid therapy (Salem et al. 1994). More recently, studies have raised concern that supplementing corticosteroids in the perioperative period may lead to poorer postoperative outcomes as it may be associated with hyperglycemia, infectious complications, venous thromboembolism (VTE), poor surgical site healing, and increased length of stay (LOS) (Mathis et al. 2004; Nguyen et al. 2014; Gribsholt et al. 2015; Toner et al. 2017).

Because clinical guidelines are lacking, physicians face the challenge of balancing the risk of adrenal insufficiency in the perioperative period with the risk of postoperative complications related to corticosteroids. Therefore, we conducted a systematic review to answer the following question: In adult patients undergoing non-cardiac non-transplant surgery and who were or are exposed to corticosteroids, does the perioperative administration of corticosteroids, compared to placebo or no intervention, reduce the incidence of adrenal insufficiency?

## Methods

### Study design and participants

We included French or English language randomized controlled trials (RCTs), cohort studies (retrospective and prospective), case studies, and systematic reviews (SRs). We included articles involving humans aged ≥ 18 years old undergoing any type of surgery except cardiac surgery and transplant surgery. We excluded articles involving participants with primary adrenal insufficiency. We included articles evaluating the effect of corticosteroid administered preoperatively, regardless of the timing of administration and the duration of the corticosteroid and if corticosteroid were administered pre and post operatively. We included articles that compare corticosteroid administration to placebo, to standard of care/conventional management, or to no intervention. Articles reporting outcomes related to adrenal crisis such as hypotension, refractory hypotension, syncope, were included. Appendix 1 presents the study inclusion criteria in Table 1.

### Information sources and search methods

We searched MEDLINE via Ovid, supplemented with a PubMed search for unindexed or in-process articles, EMBASE via Ovid, and the Cochrane Central Register of Controlled Trials. All databases were searched from 1995 to January 2017. We hand-searched the reference lists of all relevant articles. Refer to Additional file 1 for literature search strategies, Additional file 2 for screening forms, and Appendix 2 for methods of study selection, data collection process, and bias assessment methods.

### Synthesis of results

Studies review revealed a wide variability in population characteristics, types of surgery, intervention, and outcomes. Since studies’ characteristics were not conducive to quantitative analysis, we performed a descriptive analysis. Each study selected was summarized and assessed for risk of bias (refer to Appendix 4 and Additional file 3).

## Results

### Study selection

Our search strategy identified 995 unique references (refer to Appendix 3 for study flow diagram). Following independent assessment of the abstracts and titles by two authors (AB, CG), we identified 48 articles for full-text assessment. Of these, 37 did not fulfill inclusion criteria. Following discussions, the two authors were in full agreement in the selection of the studies.

### Study characteristics and results

Eleven studies met selection criteria: two RCTs, five cohort studies, and four SRs. The results are discussed herein and summarized in Additional file 4. Refer to Appendix 4 for risk of bias assessment of RCTs. Refer to Additional file 3 for the risk of bias assessment of cohort studies.

#### Randomized controlled trials

Glowniak and Loriaux (1997) published the first RCT on the subject. Eighteen patients who had been taking prednisone (mean dose of 14 mg daily) over the past 2 months and with confirmed secondary adrenal insufficiency by Cortrosyn stimulation tests were randomized to continue their usual prednisone dose and either received 100 mg of hydrocortisone (HC) before entering the operating room (OR) followed by 25 mg every 6 h for 48 h then a taper or a placebo regimen of normal saline (NS). Most subjects (*N* = 16) underwent elective moderate risk surgeries such as abdominal surgeries or total knee arthroplasty. One patient in the placebo group had a significant episode of hypotension during the operation that improved following fluid administration. One patient in the corticosteroid-treated group had acute symptomatic hypotension 2 h after surgery from excess opioid administration. During the postoperative period, the blood pressure (BP), pulse rates, and postoperative complications such as fever or infection did not differ significantly between the two groups. The results of this study are limited by the small sample size. The authors note that their study could have been larger but they chose to exclude patients with a normal Cortrosyn stimulation test as well as patients undergoing surgeries they believed were not physiologically very stressful such as transurethral biopsies of the prostate.

The second RCT, by Thomason et al. (1999), studied 20 organ transplanted patients undergoing gingival overgrowth surgery under local anesthesia. All patients were on corticosteroids for ≥ 6 months prior to OR; the average daily prednisone at time of surgery was 7.9 mg. As each patient had overgrowth requiring two surgeries, each patient acted as their own control. Immediately before surgery, patients received either intravenous HC 100 mg or placebo in random, double-blind order. There was no significant difference in BP throughout surgery and postoperatively, and in ACTH measurements on completion of surgery. The main limitation of this study is its small number of participants. The applicability of the study’s findings is also limited by the studied surgical procedure. Lastly, dental procedure under local anesthesia would generally not be considered as physiologically stressful.

#### Cohort studies

Aytac et al. (2013) retrospectively analyzed patients with ulcerative colitis (UC) exposed to corticosteroids in the previous year who had undergone a restorative proctocolectomy and categorized them into two groups based on whether they received stress-dose corticosteroids. Among patient on corticosteroids up to the surgery, doses were similar among patients. Neither the mean nor median doses were reported. One hundred milligrams of HC were administered intravenously immediately before surgery, then 100 mg dose was administered every 8 h for the first 24 h and then tapered off. Patients who were on corticosteroids until surgery received their regular corticosteroid regimen during the perioperative period. Eighty-nine patients received stress-dose corticosteroids and 146 patients did not. Stress-dose corticosteroids were more frequently administered to patients who were receiving chronic corticosteroids until the time of surgery (37.1 vs 10.3%; *p* < 0.001). Sinus tachycardia developed more frequently in patients who received stress-dose corticosteroids during surgery (21.5 vs 17.8%; *p* = 0.03). One patient in the stress-dose corticosteroid group died on postoperative day 25 because of an anastomotic leak. There was no significant difference in bradycardia, BP and in postoperative complications including surgical site infection, anastomotic leak, hemorrhage, VTE, and LOS. This study was limited by its retrospective design, no matching of patients, and small number of adverse events. Also, the use of stress-dose corticosteroids was not determined by uniform criteria as the physicians planned corticosteroids regimen at their discretion.

The following year, Lamore et al. (2014) retrospectively examined inflammatory bowel disease (IBD) patients exposed to corticosteroids within the previous year, undergoing colorectal surgery. Patients were divided into three groups at the time of surgery: [1] > 1 week of prior corticosteroid exposure, not receiving maintenance therapy (*n* = 15); [2] currently receiving budesonide (*n* = 10); and [3] currently receiving oral prednisone (*n* = 24). They received intraoperative corticosteroids, as follows: 8 patients (53%) in group 1, 7 (70%) in group 2, and 20 (83%) in group 3. Despite this being a IBD referral center, there was significant variability in postoperative GC dosing practices, particularly in patients who were receiving prednisone at the time of admission. The median intraoperative HC dose was 100 mg (range, 50–267 mg); the median total postoperative dose for the first 5 days after surgery was 485 mg (range, 50–890 mg). No patients had postoperative hemodynamic instability requiring intervention. No statistically significant difference in surgical site infection and 30-day readmission rates were detected. This study was limited by its retrospective design, no matching of patients, very small sample size, and lesser number of events. Given the small sample size, they were unable to establish a statistically significant difference in patient outcomes with and without perioperative corticosteroid exposure. The study also only evaluated in-hospital tapers, thus these findings likely underestimate the perioperative corticosteroid exposure.

Zaghiyan et al. (2011) retrospectively studied 49 consecutive corticosteroid-treated patients with IBD undergoing major colorectal surgery. Patients off corticosteroids at the time of surgery but previously treated with ≥ 5 mg of oral prednisone daily for > 1 week within the previous year were also included. Preoperative median maximum corticosteroid dose was prednisone 25 mg daily (range 5–60 mg), and the mean time from last corticosteroid dose to surgery was 4 months. Patients received either high-dose steroid (HSD group) consisting of HC 100 mg IV prior to OR then 100 mg IV every 8 h for 24 h followed by a taper, or no corticosteroids. The regimen choice was at the discretion of the surgeon. Aside from a higher incidence of tachycardia in the HDS group (82 vs 42%, *p* = 0.04), there was no significant difference in hemodynamic instability. One patient in the no corticosteroids group required a single dose of intraoperative vasopressor after aggressive beta-blockade. There was no significant difference in postoperative complications. This study was limited by its small sample size and by limitations in chart documentation as to the precise preoperative corticosteroid doses and duration between corticosteroid therapy and surgery.

A year later, the same authors published a prospective cohort study of 32 consecutive corticosteroid-treated IBD patients undergoing major colorectal surgery (Zaghiyan et al. 2012a). Patients who were on corticosteroids preoperatively received a low-dose corticosteroid regimen (LDS). This consisted of one third of the IV HC equivalent of the daily preoperative corticosteroid dose (IVED) prior to OR, then one-third IVED every 8 h for the first 24 h, followed by one quarter IVED every 8 h. Patients who had previously been treated with corticosteroids but who were not on corticosteroid therapy at the time of operation were given no perioperative corticosteroids. Patient selection for this regimen was at the discretion of the surgeon. Five patients (23%) developed intra-operative or postoperative hypotension, all in the group not taking corticosteroids at the time of surgery. In all cases, hypotension resolved either spontaneously or with fluid bolus which allowed the authors to conclude that in steroid-treated IBD patients undergoing major colorectal surgery, the use of low-dose perioperative corticosteroids seems safe. This study was limited by its small sample size and by limitations in chart documentation as to the precise preoperative corticosteroid doses and duration between corticosteroid therapy and surgery. In addition, it is difficult to comment on the clinical importance of hemodynamic instability, fever, and hypothermia and surgical outcomes without a comparison group of patients treated with high-dose corticosteroids.

Subsequently, the same team published a retrospective study of 97 IBD patients on corticosteroids or who had previously been on corticosteroids undergoing major colorectal surgery (Zaghiyan et al. 2012b). Patients received one of two perioperative corticosteroid dosing regimens: HDS or LDS regimen as described in their previous studies (Zaghiyan et al. 2011, 2012a). Patients off corticosteroids at the time of surgery who were assigned to the LDS treatment group received no perioperative corticosteroids. In patients on corticosteroids at the time of surgery (*n* = 48), there was a significantly higher incidence of overall hemodynamic instability in the LDS group (16/16, 100%) compared with the HDS group (23/32, 72%) (*p* = 0.02). However, these differences in hemodynamic instability appeared to be clinically insignificant, as in 36 of the 39 patients’ hemodynamic instability resolved with no intervention, fluid bolus, or blood transfusion. Three patients in the LDS group were treated with vasopressors. This study was limited by its small sample size and small number of events. It had a high risk of selection bias as the LDS algorithm was gradually implemented over time and was dependent on surgeon preference. At the beginning of the study period, LDS were administered to low-risk patients at the attending surgeons’ discretion, with time it was then implemented for patients on higher dose of corticosteroids.

#### Systematic reviews

A total of four SRs met our inclusion criteria. de Lange and Kars (2008) included studies analyzing the need for perioperative corticosteroid supplementation, whether these studies included a stress-dose corticosteroid regimen or not. The same year, Marik and Varon (2008) undertook a SR, including RCTs comparing stress doses of corticosteroids with placebo and cohort studies that followed patients after surgery in which perioperative stress doses of corticosteroids were not administered. Yong et al. published a Cochrane SR including only two RCTs in 2009 and then an updated review in 2012 comparing use of supplemental perioperative corticosteroids to placebo in adults on maintenance doses of corticosteroids (Yong et al. 2009, 2012). The latter review was withdrawn from publication in 2013 due to questionable eligibility criteria and interpretation of summarized evidence. In all four SRs, authors highlighted that available evidence on this topic is limited by studies with small sample sizes and flawed methodology. de Lange and Kars and Marik and Varon concluded that in patients receiving long-term adrenally suppressive doses of corticosteroids, the combination of the patient’s baseline exogenous corticosteroid plus endogenous corticosteroid production is possibly adequate to meet the demands of the physiologic stress of surgery. As for Yonge et al., given the poor level of evidence available, they decided not to support nor to refute the use of perioperative corticosteroids in patients with adrenal insufficiency. To note that all four SRs included the RCTs, we identified Glowniak and Loriaux (1997) and Thomason et al. (1999), which are reviewed in the “Randomized controlled trials” section.

## Discussion

### Summary of evidence

Though the prevalence of patients at risk of adrenal insufficiency in the perioperative period is increasing, there remains a paucity of studies on the subject (Benard-Laribiere et al. 2017; Fardet et al. 2011). Only two small RCTs (total of 37 patients) and five cohort studies (total of 462 patients) were identified for review. Neither RCT showed a significant difference in outcomes when stress-dose corticosteroids were administered compared to no perioperative corticosteroid use. This was supported by the findings of five cohort studies. The cohort studies done by Zaghiyan and colleagues in IBD patients suggest a possible benefit from receiving less corticosteroids, with HSD group reporting more tachycardia (Zaghiyan et al. 2011) and more blood loss than the LDS group (Zaghiyan et al. 2012b). Furthermore, though not included in our search due to our eligibility criteria, these results are supported by five additional cohort studies in which patients received their usual daily dose of corticosteroids without the addition of stress-dose corticosteroids; none of the patients included demonstrated biochemical evidence of adrenal insufficiency (Mathis et al. 2004; Shapiro et al. 1990; Bromberg et al. 1991, 1995; Friedman et al. 1995).

### Limitations

There have been very few studies done on the topic in the last decade, and no new RCT. As detailed above, all studies lack quality and need to be interpreted with caution. Importantly, there was significant heterogeneity within the studies in regard to both patients’ previous exposure to corticosteroids, in the stress-dose regimen itself as well as in the type of surgery. The inclusion of gingival surgery and orthopedic surgeries is debatable, as previous studies had suggested that orthopedic surgery results in less increase in cortisol levels than, for instance, abdominal surgery (Naito et al. 1991; McIntosh et al. 1981; Jasani et al. 1968). Also, most studies did not report the use of a corticosteroid taper, which could have influenced the occurrence of postoperative complications. The anesthetic agents were also not reported, and this may have an impact on hemodynamics as etomidate, a commonly used agent, inhibits the 11β-hydroxylase enzyme that converts 11β-deoxycortisol into cortisol and predictably reduces cortisol synthesis for up to 48 h after a single intubating dose of this hypnotic agent (Jackson Jr 2005; Vinclair et al. 2008).

## Conclusion

Based on the findings of this systematic review, it is not possible to conclude that the perioperative administration of corticosteroids, compared to placebo or standard of care, reduces the incidence of clinical adrenal insufficiency. Nonetheless, the above trials suggest that the demands of physiologic stress are met by a combination of increased endogenous adrenal function plus exogenous baseline doses of corticosteroids. Based on the very limited evidence available, it seems that stress-dose corticosteroid therapy in the perioperative period may therefore not be required as administration of the patient’s daily maintenance dose of corticosteroid could be sufficient in most cases. This review allows us to conclude however that the current widespread practice of perioperative supra-physiological corticosteroid supplementation in patients who have been on steroids prior to surgery is not supported by the literature. Additionally, high doses of corticosteroids are associated with important postoperative complications that should not be ignored. This further reinforces the need for a high-quality randomized control trial on perioperative corticosteroid administration.

### Additional files

Additional file 1: Literature search strategies. (DOCX 51 kb)

Additional file 2: Screening forms. (DOCX 44 kb)

Additional file 3: Risk of bias for cohort studies. Table S1. Risk of bias, confounding and precision summary: review authors’ judgements about each risk of bias for each included cohort study. (Aytac et al. 2013; Lamore et al. 2014; Zaghiyan et al. 2011, 2012a, 2012b). (DOCX 51 kb)

Additional file 4: Summary of included studies. Table S2. Studies investigating the use of supplemental perioperative corticosteroids. (Glowniak and Loriaux 1997; Thomason et al. 1999; Aytac et al. 2013; Lamore et al. 2014; Zaghiyan et al. 2011, 2012a, 2012b; de Lange and Kars 2008; Marik and Varon 2008; Yong et al. 2012, 2009). (DOCX 67 kb)

## Figures and Tables

**Fig. 1:**
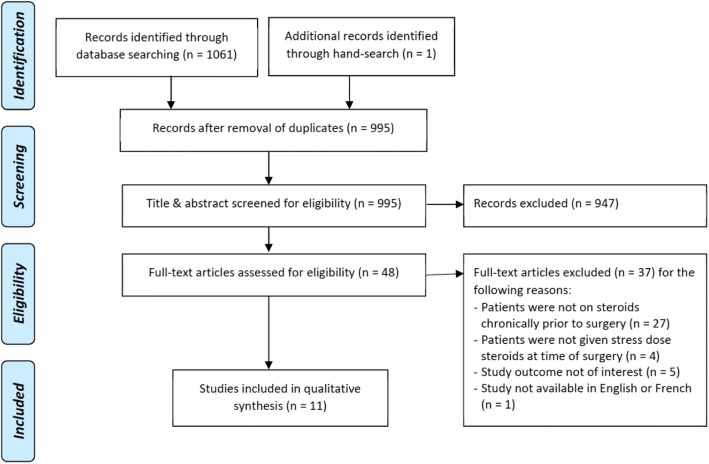
Study flow diagram

**Fig. 2:**
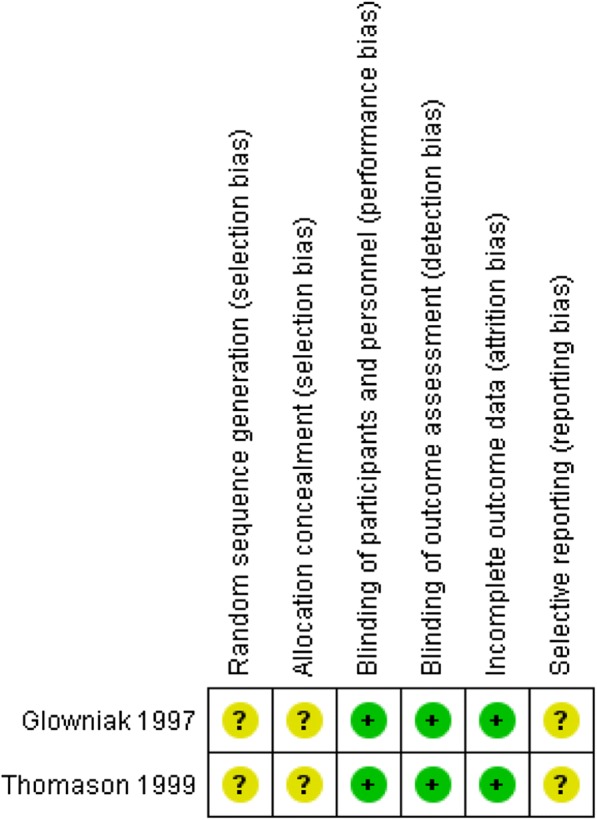
Risk of bias summary for included randomized controlled study. Green: low risk of bias; yellow: unclear risk of bias; red: high risk of bias

## Appendix 1

**Table 1:** Study inclusion criteria

Inclusion criteria	
Study designs: RCT, cohort studies, case-studies, SR	
Articles in English or French	
Population: Human participants not known to have adrenal insufficiency Age ≥ 18 years old Noncardiac nontransplant surgery	
Intervention: corticosteroid administration before and after surgery	
Comparator: standard of care, placebo, or no intervention	
Outcomes: adrenal crisis, hypotension or syncope	

*RCT* randomized controlled trials, *SR* systematic review

## Appendix 2

### Search methods

#### Study selection

Two authors (AB, CG) independently assessed the titles and abstracts of all articles identified by the above search. Any disagreements were resolved by consensus. Potentially relevant studies were subsequently retrieved in full text.

#### Data collection process

Two authors (AB, CG) independently extracted the data using a data extraction form. Any disagreements were resolved by consensus. Extracted data included study design, year of publication, surgery type, participant eligibility criteria, corticosteroid regimen used as intervention, comparative arm, sample size, primary and secondary outcomes, and results for the particular study. Studies that were found on full-text assessment not to meet inclusion criteria were subsequently excluded. Reasons for exclusion were recorded.

#### Risk of bias of individual studies

All studies meeting the inclusion criteria except for the systematic reviews were assessed for risk of bias within the individual study. Randomized controlled trials were assessed via Cochrane risk of bias tool described in the Handbook for Systematic Reviews of interventions version 5.1.0, Chapter 8 (The Cochrane Collaboration 2011). Overall risk of bias was assessed considering the risk of selection bias, performance bias, detection bias, attrition bias, and reporting bias. Each potential bias was characterized as having low, high, or unclear risk of bias. Observational studies were assessed using the agency for Healthcare Research and Quality analytic framework (Viswanathan et al. 2013). This tool was developed to consider source of confounding and risk of biases in observational studies used in systematic reviews.

## Appendix 4

### Risk of bias for RCTs

#### Risk of bias

Both studies were described by their authors as RCT; however, no information on the randomization process and on the method of concealment were described. Both studies stated patients were blinded by using a corresponding placebo. Glowniak et al. mention the surgical teams were blinded; however, the blinding of other parties is not described. No information was provided on personnel blinding in the Thomason et al.’s study. Detection and attrition bias were considered low risk in both studies as there were no differences between groups in how outcomes were determined and there were no noted withdrawals from the studies. Selective reporting bias was deemed unclear risk as the protocols of either study could not be obtained for comparison.

## Abbreviations

ACTH: Adrenocorticotropic hormone
BP: Blood pressure
CC: Case control
CD: Crohn’s disease
HC: Hydrocortisone
HDS: High dose steroids
HR: Heart rate
IBD: Inflammatory bowel disease
ICU: Intensive care unit
IV: Intravenous
IVED: Intravenous hydrocortisone equivalent of the daily preoperative steroid dose
IVF: Intravenous fluids
LDS: Low dose steroids
LOS: Length of stay
NA: Not applicable
ND: Not determined
NR: Not reported
NS: Normal saline
OR: Operation room
POD: Postoperative day
RA: Rheumatoid arthritis
RCT: Randomized controlled trial
RYGB: Roux-en-Y gastric bypass surgery
SBP: Systolic blood pressure
SR: Systematic review
UC: Ulcerative colitis
VTE: Venous thromboembolism
