# Supplementary material for: Perioperative corticosteroid administration: a systematic review and descriptive analysis
Source: Perioper Med (Lond). 2018 Jun 8;7:10. doi: 10.1186/s13741-018-0092-9 (PMC5994041; doi:10.1186/s13741-018-0092-9)
Supplement: Supplementary file 1 — Literature search strategies. (DOCX 51 kb) [file 13741_2018_92_MOESM1_ESM.docx]

## Additional file 1: Literature search strategies

Date limits (1995-present for the first run in October 2015; 2015-present for the January 2017 update) are not included in the search strategies below.

| Ovid MEDLINE Search method. **1947 to Jan 11, 2017**  **(R) In-Process & Other Non-Indexed Citations, Ovid MEDLINE(R) Daily, Ovid MEDLINE(R) and Ovid OLDMEDLINE(R)** | | |
| --- | --- | --- |
| **#** | **Searches** | **Results** |
| 1 | Preoperative Period/ or Preoperative Care/ | 65322 |
| 2 | Perioperative Period/ or Perioperative Care/ or Intraoperative Care/ or Perioperative Nursing/ | 36642 |
| 3 | Postoperative Care/ or Postoperative Period/ | 103664 |
| 4 | 1 or 2 or 3 | 185776 |
| 5 | exp *Adrenal Cortex Hormones/ | 202641 |
| 6 | exp Hydrocortisone/ or (hydrocortisone or cortef or solucortef).tw. | 79340 |
| 7 | exp Methylprednisolone/ or (methylprednisolone or Solu-medrol or Medrol).tw. | 25189 |
| 8 | Prednisone/ or prednisone.tw. | 53720 |
| 9 | exp Prednisolone/ or prednisolone.tw. | 61650 |
| 10 | exp Dexamethasone/ or (dexamethasone or decadron).tw. | 71184 |
| 11 | *Steroids/ae, ct, tu [Adverse Effects, Contraindications, Therapeutic Use] | 2941 |
| 12 | 5 or 6 or 7 or 8 or 9 or 10 or 11 | 354114 |
| 13 | 4 and 12 | 2558 |
| 14 | randomized controlled trial.pt. | 507989 |
| 15 | controlled clinical trial.pt. | 98199 |
| 16 | randomi?ed.ab. | 524337 |
| 17 | placebo*.ab. | 205523 |
| 18 | clinical trials as topic.sh. | 197834 |
| 19 | randomly.ab. | 298856 |
| 20 | trial.ti. | 201829 |
| 21 | exp Cohort Studies/ | 1807637 |
| 22 | cohort*.af. | 569672 |
| 23 | 14 or 15 or 16 or 17 or 18 or 19 or 20 or 21 or 22 | 3041370 |
| 24 | 13 and 23 | 1092 |
| 25 | exp animals/ not humans.sh. | 4853216 |
| 26 | 24 not 25 | 1069 |
| 27 | (exp infant/ or exp child/) not exp adult/ | 1659960 |
| 28 | 26 not 27 | 933 |
| 29 | limit 28 to case reports | 18 |
| 30 | 28 not 29 | 915 |

| Embase Classic and Embase Search method 1947 to 2017 January 11 | | |
| --- | --- | --- |
| **#** | **Searches** | **Results** |
| 1 | exp preoperative period/ | 258405 |
| 2 | perioperative nursing/ or perioperative period/ | 44784 |
| 3 | postoperative care/ or postoperative period/ | 252254 |
| 4 | 1 or 2 or 3 | 484844 |
| 5 | exp *corticosteroid/ | 327272 |
| 6 | Hydrocortisone/ or (hydrocortisone or cortef or solucortef).tw. | 131658 |
| 7 | Methylprednisolone/ or (methylprednisolone or Solu-medrol or Medrol).tw. | 84776 |
| 8 | Prednisone/ or prednisone.tw. | 160865 |
| 9 | Prednisolone/ or prednisolone.tw. | 119563 |
| 10 | Dexamethasone/ or (dexamethasone or decadron).tw. | 140364 |
| 11 | *corticosteroid therapy/ | 6186 |
| 12 | 5 or 6 or 7 or 8 or 9 or 10 or 11 | 670187 |
| 13 | 4 and 12 | 16442 |
| 14 | "Randomized Controlled Trial (topic)"/ | 129449 |
| 15 | placebo*.ab. | 248491 |
| 16 | (random or randomly or randomis* or randomiz*).tw. | 1177658 |
| 17 | (clinic* adj2 trial*).tw. | 399531 |
| 18 | controlled clinical trial/ or "controlled clinical trial (topic)"/ | 476330 |
| 19 | cohort analysis/ | 310739 |
| 20 | cohort*.af. | 690438 |
| 21 | 14 or 15 or 16 or 17 or 18 or 19 or 20 | 2378258 |
| 22 | 13 and 21 | 3474 |
| 23 | limit 13 to (randomized controlled trial or controlled clinical trial) | 1841 |
| 24 | 22 or 23 | 3506 |
| 25 | (animal cell/ or animal experiment/ or animal model/ or animal tissue/) not ((animal cell/ or animal experiment/ or animal model/ or animal tissue/) and human/) | 2760833 |
| 26 | 24 not 25 | 3430 |
| 27 | limit 26 to exclude medline journals | 333 |

### **Cochrane Library Search Method**

Date Run: 11/01/17 12:17:50.349

| ID | Search | Hits |
| --- | --- | --- |
| #1 | (preop* or periop* or postop*):ti,ab,kw | 82846 |
| #2 | (corticosteroid*):ti,ab,kw | 12518 |
| #3 | (hydrocortisone or cortef or solucortef):ti,ab,kw | 7581 |
| #4 | (methylprednisolone or Solu-medrol or Medrol):ti,ab,kw | 3455 |
| #5 | (prednisone):ti,ab,kw | 6483 |
| #6 | (prednisolone):ti,ab,kw | 4493 |
| #7 | (dexamethasone or decadron):ti,ab,kw | 6183 |
| #8 | (random or randomly or randomis* or randomiz*):ti,ab,kw | 565973 |
| #9 | cohort*:ti,ab,kw | 28893 |
| #10 | #2 or #3 or #4 or #5 or #6 or #7 | 34043 |
| #11 | #1 and #10 | 3103 |
| #12 | #8 and #9 | 17138 |
| #13 | #11 and #12 | 42 |

###

### **PubMed Search Method**

Date Run: 11/01/17 12:17:50.349

| Search | Query | Items found |
| --- | --- | --- |
| #7 | Search **(#5 and #6)** | 27 |
| #6 | Search **(publisher[sb] NOT pmcbook)** | 501300 |
| #5 | Search **(#3 and #4)** | 1266 |
| #4 | Search **((random*[Title/Abstract]) OR placebo*[Title/Abstract]) OR cohort*[Title/Abstract]** | 1306125 |
| #3 | Search **(#1 and #2)** | 3681 |
| #2 | Search **(((((hydrocortisone[Title/Abstract] OR cortef[Title/Abstract] OR solucortef[Title/Abstract])) OR (methylprednisolone[Title/Abstract] OR Solu-medrol[Title/Abstract] OR Medrol[Title/Abstract])) OR prednisone[Title/Abstract]) OR prednisolone[Title/Abstract]) OR (dexamethasone[Title/Abstract] OR decadron[Title/Abstract])** | 122186 |
| #1 | Search **(((preoperative[Title/Abstract]) OR perioperative[Title/Abstract]) OR intraoperative[Title/Abstract]) OR postoperative[Title/Abstract]** | 573777 |
