# Supplementary material for: Perioperative corticosteroid administration: a systematic review and descriptive analysis
Source: Perioper Med (Lond). 2018 Jun 8;7:10. doi: 10.1186/s13741-018-0092-9 (PMC5994041; doi:10.1186/s13741-018-0092-9)
Supplement: Supplementary file 2 — Screening forms. (DOCX 44 kb) [file 13741_2018_92_MOESM2_ESM.docx]

## Additional file 2: Screening forms

| **Title and Abstract Screening Form** |
| --- |
| ***Does the administration of corticosteroid prior to noncardiac surgery, compared to placebo or no intervention, reduce the incidence of adrenal insufficiency?*** |
| Articles with answer "yes or unclear" to all 5 criteria should be included. |
| If an article is excluded, check the first criteria not met that led to exclusion. |
|  |
| 1. Is this article a RCT, a cohort, a case-study, or a systematic review (SR)? |
| a. If no, exclude. |
| b. If yes or unclear, go to the next question. |
|  |
| 2. Is this article about adult human? |
| *Note: Do include article involving participants aged ≥ 18 year old.* |
| a. If no, exclude. |
| b. If yes or unclear, go to next question. |
|  |
| 3. Is this article about patient undergoing non-cardiac and non-transplant surgery? |
| *Note: Do include articles that involve patients undergoing any type of surgery except cardiac surgery and transplant surgery. Cardiac surgery is defined as any surgery that involves the heart cavity such as coronary artery bypass surgery and valve surgery. Transplant surgery is defined as any surgery that involves transplantation of kidney, liver, heart, lungs or bone marrow.* |
|  |
| a. If no, exclude. |
| b. If yes or unclear, go to next question. |
|  |
| 4. Is the article about people who received corticosteroid prior to surgery? |
| *Note: Do include articles that evaluate the effect of iron administered preoperatively, regardless of the timing of administration and the duration of the corticosteroid. Do include articles if corticosteroid was administered pre AND post. Include articles that have used any type of corticosteroid (intravenous or per os), any form of corticosteroid (Hydrocortisone (cortef or solucortef), Methylprednisolone (Solu-medrol, Medrol), Prednisone, prednisolone, dexamethasone (decadron)) and any dosage of corticosteroid.* |
|  |
| a. If no, exclude. |
| b. If yes or unclear, go to next question. |
|  |
| 5. Does the article compare the corticosteroid therapy to placebo or to no intervention? |
| Note: *Do include articles that compare corticosteroid administration to placebo or to standard of care/conventional management or to no intervention.* |
| a. If no, exclude. |
| b. If yes or unclear, include. |
| **Full Text Screening Form** |
| ***Does the administration of corticosteroid prior to noncardiac surgery, compared to placebo or no intervention, reduce the incidence of adrenal insufficiency?*** |
| Check all criteria that apply for each article. |
| If all 6 criteria are met, the article can be INCLUDED |
| If any one of the 6 criteria is not met, the study must be EXCLUDED |
| If any one of the criteria is marked as "?", the study must be classified as UNCERTAIN |
| Studies classified as UNCERTAIN will be discussed with another reviewer for consensus. |
| Studies with disagreement between reviewers will be discussed for consensus. |
|  |
| 1. Is this article a RCT, cohort, case-study, or SR? |
| a. If no, exclude. |
| b. If yes, write the type of study and go to the next question |
| c. If uncertain, add a question mark and go to the next question. |
|  |
| 2. Is this article about adult human? |
| *Note: Do include article involving participants aged ≥ 18 year old.* |
| a. If no, exclude. |
| b. If yes, add a checkmark and go to the next question |
| c. If uncertain, add a question mark and go to the next question. |
|  |
| 3. Is this article about patient undergoing non-cardiac and non-transplant surgery? |
| *Note: Do include all articles that involve patients who undergo any type of surgery except cardiac and transplant surgery. Cardiac surgery is defined as any surgery that involves the heart cavity such as coronary artery bypass surgery and valve surgery. Transplant surgery is defined as any surgery that involves transplantation of kidney, liver, heart, lungs or bone marrow.* |
| a. If no, exclude. |
| b. If yes, describe the type of surgery and go to the next question |
| c. If uncertain, add a question mark and go to the next question. |
|  |
| 4. Does the article is about people who were administered corticosteroid preoperatively? |
| *Note: Do include articles that evaluate the effect of iron administered preoperatively, regardless of the timing of administration and the duration of the corticosteroid. Do include articles if corticosteroid was administered pre AND post. Include articles that have used any type of corticosteroid (intravenous or per os), any form of corticosteroid (Hydrocortisone (cortef or solucortef), Methylprednisolone (Solu-medrol, Medrol), Prednisone, prednisolone, dexamethasone (decadron)) and any dosage of corticosteroid.* |
| a. If no, exclude. |
| b. If yes, describe the regimen(s) studied and go to the next question |
| c. If uncertain, add a question mark and go to the next question. |
|  |
| 5. Does the article compare the corticosteroid therapy to placebo or standard of care? |
| *Note: Do include articles that compare iron administration to placebo or to standard of care/conventional management or to standard intervention.* |
| a. If no, exclude. |
| b. If yes, describe the comparative arm and go to the next question |
| c. If uncertain, add a question mark and go to the next question. |
|  |
| 6. Does the article report any of the following perioperative outcomes: hypotension, refractory hypotension, hypoglycemia, syncope, or other signs or symptoms associated to adrenal crisis? |
| *Note: Perioperative refers to the periods within 48h before surgery, intra-operatively* |
| *and postoperatively (up to 6 months after surgery).* |
| a. If no, exclude. |
| b. If yes, describe outcomes and results and include. |
| c. If uncertain, add a question mark. |
