# Supplementary material for: Perioperative corticosteroid administration: a systematic review and descriptive analysis
Source: Perioper Med (Lond). 2018 Jun 8;7:10. doi: 10.1186/s13741-018-0092-9 (PMC5994041; doi:10.1186/s13741-018-0092-9)
Supplement: Supplementary file 3 — Risk of bias for cohort studies. Table S1. Risk of bias, confounding and precision summary: review authors’ judgements about each risk of bias for each included cohort study. (Aytac et al. 2013; Lamore et al. 2014; Zaghiyan et al. 2011, 2012a, 2012b). (DOCX 51 kb) [file 13741_2018_92_MOESM3_ESM.docx]

## Additional file 3: Risk of bias for cohort studies

**Table S1: Risk of bias, confounding and precision summary: review authors’ judgements about each risk of bias for each included cohort study**

| **Cohort studies**  **Questions to assess the risk of bias** | **Aytac et al,**  **2013 (11)** | **Lamore et al,**  **2014 (12)** | **Zaghiyan et al,**  **2011**  **(13)** | **Zaghiyan et al,**  **2012 (14)** | **Zaghiyan et al,**  **2012 (15)** |
| --- | --- | --- | --- | --- | --- |
| Q1 Do the inclusion/exclusion criteria vary across the comparison groups of the study? (selection bias) | Yes | Yes | Yes | Yes | Yes |
| Q2 Does the strategy for recruiting participants into the study  differ across groups? (selection bias and confounding). | No | No | No | No | Yes |
| Q3 Is the selection of the comparison group inappropriate, after taking into account feasibility and ethical considerations? (selection bias and confounding) | No | No | No | No | No |
| Q5 Was the outcome assessor not blinded to the intervention or exposure status of participants? (detection bias) | Yes | Yes | Yes | Yes | Yes |
| Q6 Were valid and reliable measures, implemented consistently across all study participants used to assess  inclusion/exclusion criteria, intervention/exposure outcomes,  participant health benefits and harms, and confounding? (Detection bias, confounding) | No | No | No | No | No |
| Q7 Was the length of follow-up different across study groups? (Attrition bias) | No | No | No | No | No |
| Q8 In cases of high loss to follow-up (or differential loss to  Follow-up), was the impact assessed (e.g., through sensitivity analysis or other adjustment method)? (Attrition bias, detection bias) | NA | NA | NA | NA | NA |
| Q12 Any attempt to balance the allocation between the groups or match groups (e.g., through stratification, matching, propensity scores)*? (*Confounding) | No | No | No | No | No |
| Q 13 Were the important confounding variables taken into account in the design and/or analysis (e.g., through matching, stratification, interaction terms, multivariate analysis, or other statistical adjustment such as instrumental variables)? (Confounding) | No | No | No | No | No |
| Q15 Are the statistical methods used to assess the primary  benefit outcomes inadequate? (Precision) | No | No | No | Yes | No |
| Q16 Are the statistical methods used to assess the main harm or adverse event outcomes inadequate? (Precision) | No | No | No | No | No |
