# Supplementary material for: Perioperative corticosteroid administration: a systematic review and descriptive analysis
Source: Perioper Med (Lond). 2018 Jun 8;7:10. doi: 10.1186/s13741-018-0092-9 (PMC5994041; doi:10.1186/s13741-018-0092-9)
Supplement: Supplementary file 4 — Summary of included studies. Table S2. Studies investigating the use of supplemental perioperative corticosteroids. (Glowniak and Loriaux 1997; Thomason et al. 1999; Aytac et al. 2013; Lamore et al. 2014; Zaghiyan et al. 2011, 2012a, 2012b; de Lange and Kars 2008; Marik and Varon 2008; Yong et al. 2012, 2009). (DOCX 67 kb) [file 13741_2018_92_MOESM4_ESM.docx]

## Additional file 4: Summary of included studies

| **Table S2: Studies investigating the use of supplemental perioperative corticosteroids** | | | | | | | | |
| --- | --- | --- | --- | --- | --- | --- | --- | --- |
| **Source** | **No. of pts** | **Design** | **Patient description** | **Previous corticosteroids** | **Surgical procedure** | **Corticosteroid Regimen** | **Comparator** | **Result** |
| Glowniak et al,  1997 (9) | 6/12^1^ | RCT  Blind | Various | 12 mg/2 y  (corticosteroid)  16 mg/5 y^3^  (placebo) | Various non-cardiac,non-transplant surgery | Usual daily dose with HC 100 mg IV at OR then 25 mg q 6 h for 48h | Usual daily dose with NS placebo regimen | In cortisol group, 1 patient had symptomatic hypotension (74/50 mmHg) (thought to be due to excess opioid and volume depletion).  No significant differences (tachycardia, infection, ileus). |
| Thomason et al, 1999 (10) | 20/40^2^ | RCT  crossover design | Organ transplant | 7.9 mg/3 y^3^ | Gingival overgrowth surgery | Usual daily dose with HC 100 mg at OR | Usual daily dose with NS placebo regimen | No hemodynamic difference  No ACTH measurement difference |
| Aytac et al, 2013 (11) | 89/ 146^1^ | R cohort | UC | NR | Restorative procto-colectomy (RP) | HC 100 mg IV before surgery in the OR, then HC 100mg IV 1 q 8h x 24hrs, then taper | Pts under steroids until RP received their regular steroid regimen.  Pts not treated with steroid preop received nothing. | HR >100: 17.8% (no stress dose) vs 21.5% p = 0.03  HR <60, SBP <90, SBP >140, SBP< 90, Temperature: no difference  Complications (infection, VTE, adrenal insufficiency, LOS, reoperation): no difference |
| Lamore et al,  2014 (12) | 35/14^1^ | R cohort | IBD | NR | Major abdominal surgery | Median intraoperative HC dose was 100 mg (range, 50-267 mg) Median total postoperative dose for the first 5 days after surgery was 485 mg (range, 50-890 mg). | Pts under steroids until OR received their regular steroid regimen.  Pts not treated with steroid preop received nothing. | Surgical site infection: no difference  30-d readmission: no difference  No difference within 3 steroid groups  . |
| Zaghiyan et al,  2011  (13) | 11/38^1^ | R cohort | IBD | 25mg/day (HDS) vs 40mg/day (NS) ^5^ | Major colorectal surgery | HC 100 mg IV at OR then 100 mg IV q 8h for 24h then taper prednisone 20 mg die over next 3 days. | No steroids | HR >100: 82% (HDS) vs 42% (NS) p = 0.04  HR <60, SBP <90, temperature, hemodynamic instability: no difference  Complications (minor or major surgical): no difference |
| Zaghiyan et al,  2012 (14) | 10/22^1^ | P cohort | IBD | 23 mg/day for patients taking steroids at the time of surgery vs 40 mg/ day ^4^ (NS) | Major colorectal surgery | 1/3 IV HC equivalent of baseline dose at OR then 1/3 of baseline dose q 8h, then 1/4 of baseline dose q 8h, then 1/6 of baseline dose q 8h, then q 12h. | No steroids | HR > 120: 40% (LDS) vs 5% (NS).  5 pts (23%, NS) developed intra-operative or postoperative hypotension (SBP < 90 mmHg), all in the group that was not taking steroids at the time of surgery  No significant difference in postop outcomes and overall surgical morbidity |
| Zaghiyan et al,  2012 (15) | 43/54^1^ | R cohort | IBD | 20 mg/day (HDS) vs 38 mg/day (LDS)^6^ | Major colorectal surgery | HC 100 mg IV at OR then 100 mg IV q 8h for 24h then taper prednisone 20mg die over next 3 days. | 1/3 IV HC equivalent of baseline dose at OR, then 1/3 of baseline dose q 8h, then 1/4 of baseline dose q 8h, then 1/6 of baseline dose q 8h, then q 12h. | No significant difference between 2 groups except intraoperative blood loss more in HDS (150cc vs 100cc, p=0.02). Outcomes assessed: IV fluid requirement, transfusion, surgical complications, LOS).  Hemodynamic instability in patients on steroids at the time of surgery: 72% (HDS) vs 100% (LDS), p=0.02 🡪 clinically insignificant (no IVF, transfusion, or intervention required) |
| De Lange et al,  2008 (16) | NA | SR | RCT (2),  R cohort(2) P cohort(3) | NA | All types of surgeries | NA | NA | Lack of evidence  No significant differences in the outcomes (adrenal insufficiency, hypotension). |
| Marik et al,  2008 (17) | NA | SR | RCT (2),  P cohort (6) R cohort(1) | NA | Various | NA | NA | Among RCTs: no SBP difference between groups  In cohort studies that continued steroids periop (no stress dose): no hypotension and no AI.  In cohort studies that stopped usual dose of steroid preop (at least 48-36 hours preop): 2 patients had hypotension that responded to fluid and steroids. |
| Yong 2009 ,2012 (18, 19) | NA | SR | RCT (2) | NA | Various | NA | NA | Lack of evidence  No significant differences in the outcome (mortality, LOS, hypotension). |
| Abbreviations: ACTH: Adrenocorticotropic hormone; AI: Adrenal insufficiency CC: case-control; H: Hours; HC: Hydrocortisone; HDS: High dose steroid; HR: Heart rate; IBD: Inflammatory bowel disease; IV: Intravenous; IVF: Intravenous fluid; LDS: Low dose steroid; LOS: Length of stay; NA: Not applicable; ND: Not determined; NR: Not reported; NS: Normal saline; OR: Operation room; P: Prospective; R: Retrospective; RA: Rheumatoid arthritis; RCT: Randomized controlled trial; SBP: Systolic blood pressure; SR: Systematic review; UC: Ulcerative colitis  Indicates number of treatment/control patients ^2^ Indicates the number of patients/surgical procedures ^3^ Reported as mean daily dose of prednisone/duration ^4^ 40 mg/day is the median dose of maximum steroid therapy within the past year for patients who were not the taking steroids at the time of operation ^5^ Preoperative maximum steroid dose. Duration not reported. ^6^ Steroid dose at time of surgery. Duration not reported. | | | | | | | | |
